# Experiences, Challenges, and Lessons Learned During Implementation of a Remote Monitoring Program for Home-Isolated COVID-19 Patients in Chennai, India

**DOI:** 10.9745/GHSP-D-21-00458

**Published:** 2023-02-28

**Authors:** Parasuraman Ganeshkumar, Kumaravel Ilangovan, M. Jagadeesan, Madhusudhan Reddy, Fermi P. Vidhya, Vignesh Vairamani, Hemalatha Masanam Sriramulu, S. Banumathi, Prakash Govindasamy, Prabhdeep Kaur

**Affiliations:** a National Institute of Epidemiology, Indian Council of Medical Research, Chennai, India.; b Greater Chennai Corporation, Government of Tamil Nadu, Chennai, India.

## Abstract

During the early months of the COVID-19 pandemic in 2020, the majority of the identified COVID-19 patients in Chennai, a southern metropolitan city of India, presented as asymptomatic or with mild clinical illness. Providing facility-based care for these patients was not feasible in an overburdened health system. Thus, providing home-based clinical care for patients who were asymptomatic or with mild clinical illnesses was a viable solution. Because of the imminent possibility of worsening clinical conditions in home-isolated COVID-19 patients, continuous monitoring for red flag signs was essential. With growing evidence of the effectiveness of remote monitoring of patients, the Greater Chennai Corporation in partnership with the National Institute of Epidemiology conceptualized and implemented a remote monitoring program for home-isolated COVID-19 patients. The key steps used to develop the program were to (1) decentralize triage systems and establish a home-isolation protocol, (2) develop a remote monitoring platform and remote health care workforce, and (3) onboard patients and conduct remote hybrid monitoring. In this article, we share the pragmatic solutions, critical components of the systems and processes, lessons, and experiences in implementing a remote monitoring program for home-isolated COVID-19 patients in a large metropolitan setting.

## Abstract (precis)

We describe the lessons learned from developing and implementing a remote monitoring program for home-isolated COVID patients in a limited-resource setting.

## Teaser

**Key Findings:**

- The stress of the COVID-19 pandemic on the health system in Chennai, India, necessitated the development of a remote monitoring program for home-isolated patients.
- Home-isolation care for COVID-19 patients warranted suitable home conditions, standardized data collection mechanisms, robust systems and processes for early identification of clinical deterioration, and prompt referral to facility-based care.

**Key Implication:**

- The processes we describe can be adapted and contextualized for use in other limited-resource settings to provide clinical care and social support for home-isolated COVID-19 patients.

## INTRODUCTION

The COVID-19 pandemic continues to pose challenges for global public health and all economies worldwide.^1^ At the height of the pandemic in October 2020, India and one of its southern states, Tamil Nadu, had reported 8,184,082 and 38,093 active cases, respectively.^2^

The clinical presentation of COVID-19 varies from asymptomatic to severe symptoms. Asymptomatic COVID-19 patients and patients with mild clinical features at the time of laboratory confirmation may deteriorate clinically to moderate and severe over time.^3^^–^^6^ Patients with asymptomatic and mild symptoms can recuperate at their homes with the appropriate care and monitoring, which helps break the chain of transmission, reduce exposure for health care workers, and optimize health system resources.^3^^,^^7^^–^^9^

After Chennai reported its first COVID-19 case, the city corporation began to refer all lab-confirmed patients to 4 government-operated tertiary care COVID-19 designated hospitals for triage, treatment, and care. This referral mechanism delayed the triage process because of long patient waiting times and overwhelmed these hospitals’ non-COVID-19 care health services. Understanding the burden that COVID-19 patient care had on already overwhelmed health facilities, governments and health organizations issued guidelines for home isolation of presymptomatic, asymptomatic, and mild symptomatic COVID-19 patients.^10^^–^^13^ Then, in May 2020, Chennai city corporation initiated a system for remotely monitoring asymptomatic and mild-symptomatic home-isolated COVID-19 patients.

Monitoring home-isolated COVID-19 patients requires additional workforce and patient compliance. Remote patient monitoring is a resource-intensive, tedious, and laborious exercise for an already strained health system.^14^ However, providing clinical care in a facility setting to a large number of lab-confirmed COVID-19 patients in high-transmission settings like Chennai was not feasible at the height of the pandemic. A remote monitoring program for home-isolated COVID-19 patients had to have the ability to provide prompt triage and clinical evaluation of patients, as well as evaluation of their home care setting, monitoring of their clinical course, and immediate referral, if required. To ensure the program’s successful outcome and reduce disease transmission, community engagement and participation would also be essential to help address appropriate preventive behaviors and practices and ensure compliance and sustainability.^8^^,^^12^^,^^15^ These requirements and challenges were the impetus to find an appropriate and innovative solution for the remote monitoring of home-isolated COVID-19 patients. A remote monitoring program would enable early identification of deterioration of the patient’s health status and allow appropriate referral and care and, in turn, would help optimally use health system resources and reduce the burden on health care facilities during the pandemic.^16^^–^^18^ In this article, we share lessons learned and experiences of implementing a remote monitoring program for COVID-19 patients self-isolating at home in Chennai, India.

## REMOTE MONITORING PROGRAM FOR COVID-19 HOME-ISOLATED PATIENTS

### Setting

Chennai, the capital of Tamil Nadu in southern India, spans more than 426 km and has a population of 8.2 million. The city is divided into 3 revenue administrative regions (North, Central, and South) with 15 revenue zones and 200 divisions (wards) within the regions.

### Program Design and Development Process

**FIGURE 1:**
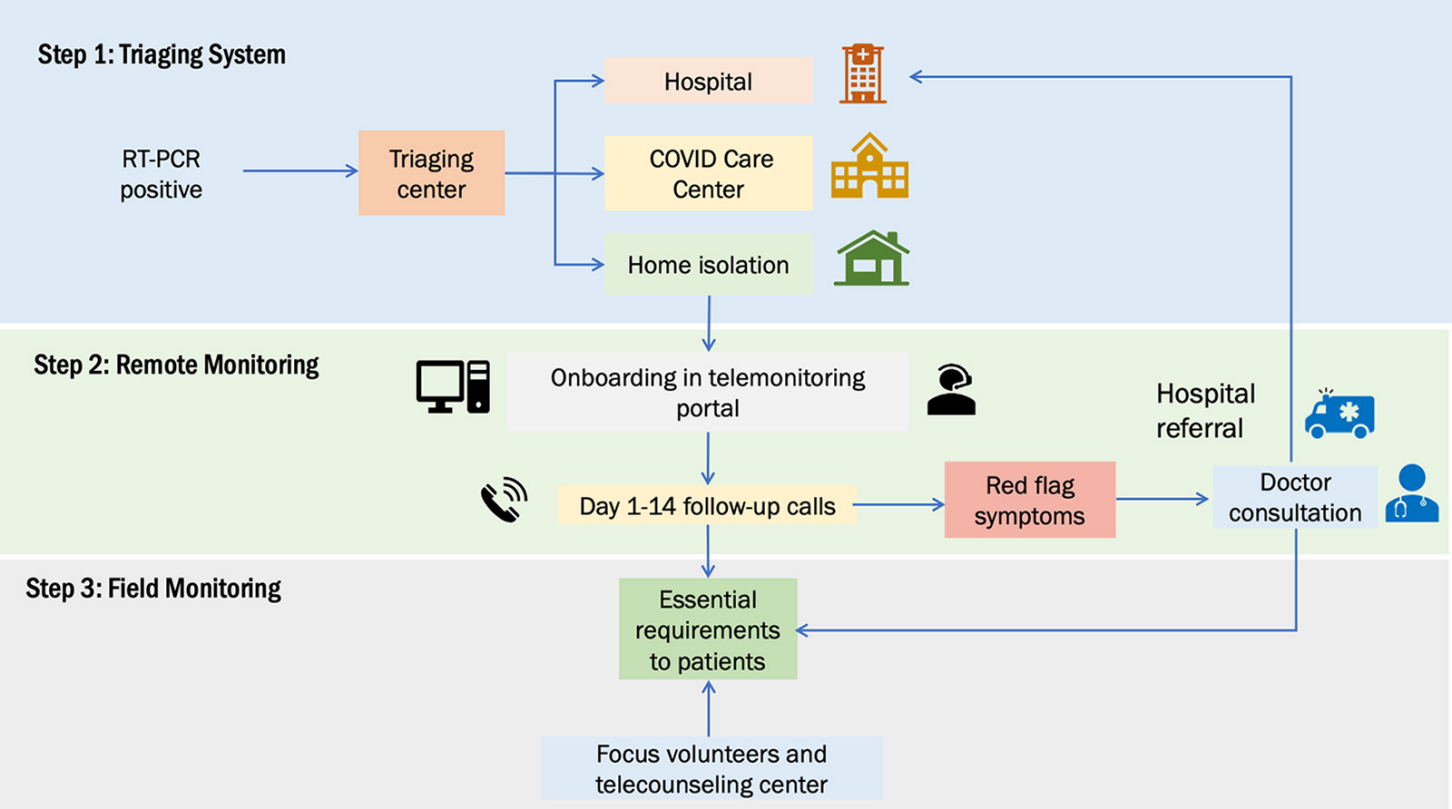
Triage and Monitoring Protocol for Remote COVID-19 Patient Monitoring Program in India Abbreviation: RT-PCR, reverse transcription-polymerase chain reaction.

With technical assistance and support from the Indian Council of Medical Research-National Institute of Epidemiology, the Greater Chennai Corporation designed a hybrid remote monitoring program that would use information communication technology and an in-person workforce to provide comprehensive medical and psychosocial support services for home-isolated COVID-19 patients.^19^ The program consisted of patient triage, daily monitoring of symptoms, counseling, doctor consultations, delivery of essential medicines, emergency ambulance services, and referral, if required. We describe the following steps used to develop the remote monitoring program (Figure 1).

#### Step 1: Decentralize Triage System and Establish Home-Isolation Protocol

**FIGURE 2:**
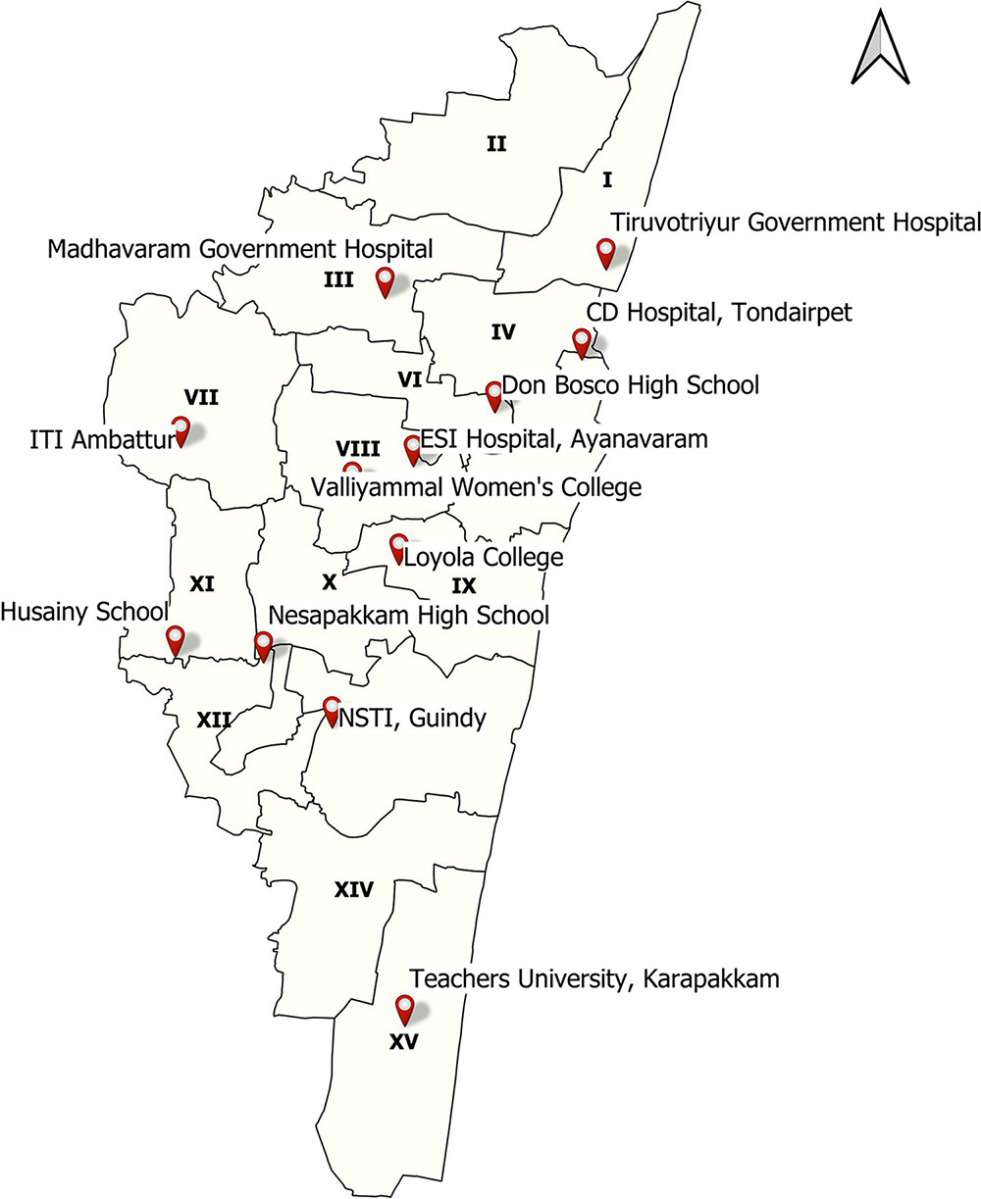
Map of COVID-19 Triage Centers in the 15 Revenue Zones of Chennai, India Abbreviations: CDH, Communicable Disease Hospital; ESI, Employee State Insurance Hospital; ITI, Industrial Training Institute; NSTI, National Skill Training Institute.

To more effectively manage patients with COVID-19 and alleviate long patient waiting times at the 4 government-operated hospitals, decentralizing the triage of COVID-19 patients was essential.^20^ To serve the entire Chennai population, we established 12 triage centers (5 in the North region, 4 in the Central region, and 3 in the South region) across the 15 revenue zones of Chennai in May 2020 (Figure 2).

To more effectively manage patients with COVID-19 and alleviate long patient waiting times at the hospitals, decentralizing the triage of COVID-19 patients was essential.

After patients were notified of their positive RT-PCR (reverse transcription-polymerase chain reaction) result, the local health official first used a standard checklist to document whether the patient’s home was fit for home isolation care and then assisted with transporting the patient by ambulance to the nearest triage center designated for the patient’s residential area (ward). At the triage center, physicians conducted a clinical evaluation of patients that included identifying the presence of any comorbidities (such as hypertension and diabetes), testing oxygen saturation, measuring complete blood count with an autoanalyzer, and performing chest X-ray imaging (if applicable). Based on the patient assessment and clinical parameters, physicians categorized patients into mild, moderate, and severe categories according to the government guideline.^21^ Patients aged younger than 60 years with asymptomatic or mild clinical features with suitable home isolation environments were referred for home isolation care with a signed undertaking form. The patients referred for home care were provided with COVID-19 kits containing gloves, masks, hand sanitizer, yellow bags for biomedical waste, paracetamol and vitamin C tablets, Kabasura Kudineer (an indigenous herbal medicinal powder used to treat hyperthermia), and pamphlets detailing the precautionary measures that patients and caregivers should practice during home isolation. The remaining patients were sent for facility-based care or to COVID-designated hospitals. Each patient was advised to sign an undertaking form to follow the home isolation care guidelines issued by the Tamil Nadu state government.^13^^,^^22^

#### Step 2: Develop a Remote Monitoring Platform and Remote Health Care Workforce

With the assistance of a software development team, we created a web-based remote monitoring platform. Details of all patients referred from triage centers for home isolation were digitized into a database, which was uploaded daily onto the remote monitoring platform. The platform enabled health care workers to contact patients over the phone for initial registration, schedule patients’ follow-up daily phone calls with health care workers, and facilitate doctor consultations when a patient’s case was escalated. The platform also stored recordings of phone conversations between patients and health care workers.

The web-based remote monitoring platform enabled health care workers to call patients for registration, schedule follow-up calls, and facilitate doctor consultations.

Identifying the remote health care workforce was challenging, as a minimum of 150 workers were required to operate the remote monitoring program effectively. The city corporation posted an online job description inviting people to apply. Having a laptop with a working Internet connection was a prerequisite to employment. We hired 321 applicants and trained them virtually because of the complete lockdown of the city and the unavailability of public transport. We also trained a team of workers to supervise and monitor newly recruited workers. Phone calls were audited for completion, quality of conversation, and recording patient data on the portal. Health care workers requiring additional support were provided with refresher training.

We prepared a standard operating procedure regarding home isolation care, the roles of each workforce member, and linkages to the remote monitoring team. Health care workers received training on using the platform, using the standard checklist of COVID-19 red flag signs and symptoms, counseling patients and their caregivers on appropriate behavior and precautions while sick, providing psychosocial support, providing nutrition advice, referring patients warranting immediate referral, and the COVID-19 testing protocol.

#### Step 3: Onboard Patients and Conduct Remote Hybrid Monitoring

Trained workers called each home-isolated patient or caregiver listed in the platform to register them in the remote monitoring program. The onboarding procedure consisted of 2 steps. First, health care workers confirmed the availability of the patient, rechecked the eligibility criteria, recorded the self-reported clinical symptoms, and obtained their oral consent for regular follow-up. Then, health care workers educated patients and caregivers on proper nutrition, precautions, and preventive care during home isolation. After onboarding patients, health care workers contacted the patients/caregivers daily to inquire about red flag symptoms and provide counseling.

If a patient reported any red flag symptoms, such as persistent fever, cough, or breathlessness, the health care worker escalated the call to the doctor. After the doctors confirmed their symptoms, and if the patient’s condition warranted it, patients were transported by ambulance to a designated hospital. Greater Chennai Corporation established a full-time COVID-19 Control Room where an emergency team consisting of 3 doctors and 12 volunteers coordinated the hospital admissions of any patients referred from home isolation.

If the patient’s condition did not warrant hospital care, the doctor prescribed medications and advised them to continue the home isolation care. If a patient was unreachable for onboarding and follow-up calls, the health care worker forwarded the request to the respective ward health officer for a home visit. Ward health officers reported the patient’s health status after physical verification. The health care workers ended the home isolation care after 10–14 days when patients met the discharge criteria. A patient who did not have symptoms, irrespective of medications, for 3 consecutive days in the 10-day isolation period from the date of notification, was considered a successful discharge.^10^^,^^23^

If a patient was unreachable for calls, the health care worker forwarded the request to the respective ward health officer for a home visit.

Greater Chennai Corporation deployed a dedicated team of staff nurses, urban health nurses, auxiliary nursery midwives, Anganwadi workers, and 200 medical officers for each division across the city to conduct home visits. Team members were equipped with a medical kit consisting of face masks, prescribed tablets, and pulse oximeters. The teams monitored home-isolated patients’ vital signs once every 2 days and provided the prescribed pills and masks if patients had not received them earlier in the triage center.^24^

In addition to providing these essential medical services, the program provided social support to patients. The Greater Chennai Corporation recruited 3,500 volunteers across 200 divisions, called FOCUS (Friends of COVID Persons Under Surveillance), to provide social support to patients, such as by purchasing groceries and other domestic essentials. Each FOCUS volunteer supported home-isolated patients in 5–10 streets to provide essential domestic services at the doorstep.^25^^,^^26^ The health care workers also provided grievance redressal support to FOCUS volunteers if any patient raised a query on medical or domestic essential services and later coordinated with the respective health care worker team to resolve the issue.

## PROGRAM RESULTS

In our program, between May 14 and October 27, 2020, 56,046 home-isolated COVID-19 patients were successfully onboarded by the health care workers. Of these, 32,068 (58%) were men and 24,206 (48%) patients were aged 30–49 years (Table 1). Health care workers made 268,567 calls to 56,046 patients during the observation period for 10 days from the date of notification of lab confirmation of COVID-19.

**TABLE 1.** Demographic Characteristics of Onboarded Home Care COVID-19 Patients in Chennai, India, May 14–October 27, 2020

**Age, Years**	**Men, No. (%)**	**Women, No. (%)**	**Total**
9 and younger	1,025 (52)	949 (48)	1974
10–29	9656 (56)	7,457 (44)	17113
30–49	14,124 (58)	10,082 (42)	24206
50–69	6,523 (57)	4,906 (43)	11429
70–89	729 (56)	566 (44)	1295
90 and older	11 (38)	18 (62)	29
Total	32,068 (57)	23,978 (43)	56046

Each patient’s outcome status was recorded on the remote monitoring platform. Between May 14 and October 27, 2020, 1,697 patients (3%) were referred to facility care or a designated COVID hospital after doctor consultations (Table 2). Because they had an unsuitable home isolation environment, 774 (1.4%) patients were transferred to COVID care centers. Of the patients referred to the hospital, 1,091 (64%) were males and the majority (42%) were aged 30–49 years (Table 3).

**TABLE 2.** Outcome of Home Care COVID-19 Patients in Chennai, India, May 14–October 27, 2020

	**Discharged**		**Facility Isolation Care and Hospital Referral**	
**Month**	**Men, No. (%)**	**Women, No. (%)**	**Men, No. (%)**	**Women, No. (%)**
May	281 (67)	141 (33)	23 (70)	10 (30)
June	4,545 (60)	3,025 (40)	176 (64)	98 (36)
July	8,113 (58)	5,850 (42)	179 (62)	112 (38)
August	5,233 (56)	4,120 (44)	235 (63)	140 (37)
September	5,671 (55)	4,581 (45)	243 (66)	125 (34)
October	6,354 (56)	5,029 (44)	246 (69)	110 (31)
Total	30,197 (57)	22,746 (43)	1,102 (65)	595 (35)

**TABLE 3.** Demographic Characteristics of Home Care COVID-19 Patients Referred to Hospitals in Chennai, India, May 14–October 27, 2020

**Age, Years**	**Men, No. (%)**	**Women, No. (%)**
9 and younger	9 (43)	12 (57)
10–29	206 (58)	149 (42)
30–49	461 (65)	253 (35)
50–69	351 (70)	151 (30)
70–89	68 (70)	29 (20)
90 and older	5 (83)	1 (17)
Total	1,091 (64)	591 (35)

Initially, we engaged doctors in all 3 processes (onboarding, escalation calls, and end isolation) of telemonitoring program consultations. However, beginning June 15, 2020, doctor consultations were reserved for escalation calls alone due to high case numbers. The escalation calls were triggered by the health care workers either based on the patient’s request or by checklist verification of the patient’s health status. Doctors conducted a total of 19,406 consultations: 5,748 (30%) between May 14 and June 15, 2020, and 13,658 (70%) between June 16 and October 31, 2020.

## IMPLEMENTATION CHALLENGES AND SOLUTIONS

While deploying the remote monitoring home isolation care monitoring program for COVID-19 patients in Chennai, we faced many implementation challenges for which we derived solutions based on stakeholders’ meetings, observations, and interactions with health care workers and doctors.

### Home Isolation and Discharge Criteria

Because of the difficulties in establishing the first symptom onset date, we considered the RT-PCR positive result notification date as day 1 for home isolation. We ensured home isolation care for 14 days. Not having any symptoms for 3 consecutive days at the end of the home isolation period was considered a criterion for discharge; however, ensuring patients were symptom free was a challenge. Receiving confirmation from an in-person visit with recorded data helped overcome this challenge.

### Patients With Unsuitable Home Isolation Care Setting

Patients triaged for home isolation care who did not have a suitable home isolation environment received intermediate care at facilities called COVID care centers. Staff nurses, medical officers, and other support staff monitored the patients at the COVID care center daily, and daily meals were provided free of cost for the entire isolation period.

### Lack of Patient Compliance

Despite signing the undertaking form and receiving counseling on precaution practices and conducting community awareness initiatives about the monitoring program, some patients violated the isolation norms.^27^ In response, trained staff counseled these patients and allowed them to isolate at the nearest COVID care centers.

Despite receiving counseling on precaution practices, some patients violated the isolation norms.

### Drug Issuance to Patients During Home Isolation Care

Management of the delivery of drugs prescribed by the doctors to home-isolated patients was done using a mobile phone message group that included doctors, volunteers, and the remote health care workforce. Physicians posted prescriptions and drug details for each patient to the group, and volunteers tagged the specific remote health care team. The message group allowed for the immediate sorting of drug requests and delivery to the patients to their homes.

### Limitations of Using New Technology

We observed a lack of knowledge about using new technology platforms by doctors and health care workers. In response, we provided focused training to use the platform and technical support around-the-clock if the users faced any hurdles. Our platform only onboarded the patients with an active mobile phone connection, which meant that the remote health workforce team had to monitor those patients without a mobile number through home visits.

### Complete Quality Data

Capturing real-time quality data of onboarding, follow-up calls, and doctor teleconsultations in standard electronic formats programmed into the portal played a crucial role in our program. Completeness of all data fields was ensured before uploading the data to the platform. Thus, we removed the ambiguity related to the discharge date and follow-up duration.

## LESSONS LEARNED

We developed and implemented a remote monitoring home isolation care program for COVID-19 patients in a large metropolitan city in south India. During our experience with the COVID-19 response, we learned several critical lessons in delivering pragmatic solutions during a pandemic.

Despite the structural limitations experienced during the pandemic, subnational governments across India responded quickly and innovatively to face this unprecedented crisis. One such innovative practice was providing home-based care to a large number of patients. The pandemic and subsequent lockdown provided the impetus for digital innovation in several sectors, including health care.^28^ Many policies and programs, such as the Union Government’s Ayushman Bharat Digital Health Mission, National Telemedicine/Consultation Services (eSanjeevani platform), and Telemedicine Practice Guidelines (March 2020), were put in place to build the telemedicine infrastructure in the country.^29^ As of September 29, 2022, through eSanjeevani, 64,504,529 individuals have received cost-free doctor consultations for various acute and chronic ailments across the country.^30^ The Indian telemedicine market is expected to exhibit a compound annual growth rate of 30.20% from 2022 to 2027.^31^ The rise of artificial intelligence, related health technologies, and widening smartphone-based Internet penetration across the country helped pave the way for telemedicine to become part of mainstream health care services.

Reviewing the remote patient monitoring programs elsewhere in India, we found that most states and a few municipal corporations adopted information and communication tools to connect and monitor home isolation patients.^28^ However, the linkages between the health care worker team and the remote in-person monitoring team were unique in our home isolation monitoring program. We could not compare our program with other programs due to scarce and incomplete information on remote home monitoring programs implemented by municipal corporations.^28^ A study conducted in Jodhpur, India, stated that patients isolated in the home had a better quality of life than those who received facility-based care.^32^ As the number of patients increased due to the high transmission of COVID-19, requiring home isolation care was necessary to protect the overwhelmed health system. Over time, when the number of patients under home isolation care increased, we felt the need for a more robust patient care solution.

The linkages between the health care worker team and the remote in-person monitoring team were unique in our hybrid home isolation monitoring program.

In our experience, we found remote monitoring to be a viable solution despite operating in a limited-resource setting. Remote monitoring as a solution for patient care in home isolation was well received by the patients and providers. A strong political will to provide quality remote health care drove us to innovate this solution. The use of technology enabled the health care system to optimize the usage of limited resources and has greatly reduced the patient care turnaround time. Documentation of patient care using our remote monitoring platform has allowed the health care system to efficiently audit its quality of care. Improved information communication systems enabled the stakeholders to provide prompt patient care and reduced the strain on the health system. Our experiences suggest that implementing a remote monitoring program for home-isolated COVID-19 patients can improve patient safety and quality of care.

## Notes

**Peer Reviewed**

**First published online:** January 18, 2023.
